# Sleep Interventions and the Prevention of Pediatric Anxiety: A Systematic Review

**DOI:** 10.1007/s11920-026-01701-4

**Published:** 2026-07-13

**Authors:** Katherine M. Kidwell, Alison Vrabec, Lyric K. Tully

**Affiliations:** Department of Psychology, Syracuse University, 352 Marley Education Center, Syracuse, NY 13224 USA

**Keywords:** Sleep intervention, Anxiety prevention, Insomnia, Adolescence, Anxiety sensitivity, Cognitive behavioral therapy

## Abstract

**Purpose of Review:**

Anxiety disorders are prevalent in children, adolescents, and young adults. As many youth either do not receive adequate intervention or do not achieve remission, prevention of anxiety disorders is a public health priority. Sleep problems are a modifiable risk factor that precedes anxiety. This systematic review includes recent pediatric sleep interventions and anxiety outcomes, and introduces anxiety sensitivity as an understudied mechanism.

**Recent Findings:**

In 21 intervention and experimental studies, sleep intervention reduced anxiety symptoms/disorders and the onset of anxiety disorders. Three additional recent studies examined anxiety sensitivity in the relationship between sleep problems and anxiety, supporting the inclusion of anxiety sensitivity in future research studies.

**Summary:**

Sleep health is a scalable, accessible target for reducing anxiety and is an important part of pediatric anxiety prevention. Sleep treatments can be brief and implemented in psychiatric practice. Future research studies should consider anxiety sensitivity as a potential mechanism.

Anxiety disorders are common in childhood and adolescence [1] and often persist into adulthood [2]. Although evidenced-based anxiety treatments exist, access and availability is limited, resulting in many young people not receiving adequate treatment. Even among individuals who do receive anxiety treatment, half do not have symptom remission following treatment receipt [3, 4]. Thus, identifying methods of anxiety *prevention* that are scalable and accessible is important. Although sleep health is not often a standard component of pediatric anxiety prevention efforts, improving sleep could be a high yield target for anxiety prevention.

Sleep problems tend to worsen throughout development [5], and include concerns such as insufficient sleep duration, frequent wakings, and clinical disorders (e.g., insomnia, breathing disorders, sleep-related movement disorders). Sleep interventions can be quickly and easily implemented in many settings, including brief sleep treatment within primary care and specialized health care. Stigma is a common barrier among youth to seeking treatment for anxiety symptoms [6], while sleep tends to be a less stigmatized topic.

Numerous longitudinal studies indicate that poor sleep at an initial time point predicted higher anxiety at a later point. Studies examining infancy, preschool, and school-aged children found that sleep problems reliably predicted the development of anxiety symptoms/disorders later in childhood/adolescence [7–9]. The sleep-anxiety relationships are also supported in adolescence and young adulthood, with research finding that sleep disturbances at age 15 predicted anxiety disorder diagnoses at ages 17, 21, and 24, over and above earlier anxiety symptoms [10]. In studies of specific sleep disorders (i.e. insomnia), baseline insomnia predicted new onset or a higher likelihood of an anxiety disorder at follow-up [11, 12]. Because longitudinal studies consistently find that sleep problems precede anxiety, sleep health is an excellent prevention target.

Anxiety sensitivity is defined as the tendency to fear physical or cognitive sensations associated with anxiety [13]. Individuals with high anxiety sensitivity are more likely to interpret somatic symptoms as dangerous, negative, or catastrophic [14]. In youth, anxiety sensitivity relates to greater instances of anxiety disorders [15] and increased sleep problems [16], including longer sleep onset latency [17], more insomnia symptoms [18], and more fatigue and nightmares [19]. Anxiety sensitivity is one connection between sleep and anxiety that may also be an easily accessible target for anxiety prevention.

We reviewed the current literature on sleep-focused interventions and anxiety outcomes in youth between the ages of 6 and 25 years. Two central questions guided the review: (1) Does improving sleep reduce anxiety symptoms/disorders or anxiety onset in young people? (2) What potential role does anxiety sensitivity serve? The primary goal of the review was to identify if there is sufficient rigorous support to recommend sleep as a prevention method of the development of anxiety disorders in youth.

## Methods

The review was preregistered through the Open Science Framework (osf.io). Using standard guidelines [20, 21], we conducted systematic searches of PsycINFO, Scopus, and CINAHL and imported articles to Rayyan.ai for de-duplication and manual screening. Search terms included: (1) sleep terms (e.g., sleep*, insomnia, “sleep problems,” “sleep quality,” “sleep intervention,” “CBT-I”); (2) anxiety terms (e.g., “anxiety disorder,” “generalized anxiety,” “social anxiety”); and (3) population terms (e.g., adolescen*, teen*, youth, child*, “young adult,” “emerging adult”). A secondary targeted search added “anxiety sensitivity” or “ASI”. Hand-searching of reference lists was also conducted.

Eligible studies included participants between 6 and 25 years (not exclusive), examined sleep interventions or utilized sleep restriction/extension experimental designs, and reported an anxiety outcome. The search was restricted to articles published from 2010 to 2026 to identify recent articles (past 5 years), as well as, older studies due to a small literature base. The abstract had to be available to review in English. Studies on anxiety sensitivity were included if they had measures of both sleep and anxiety. Studies that were exclusively with samples with autism spectrum disorder, intellectual disability, posttraumatic stress disorder, or exclusively physical health conditions (e.g., cancer) were excluded. Three reviewers screened studies for inclusion and disagreements were resolved through discussion.

## Results

### Descriptives

Table 1 contains descriptives on each included study. The review included 24 articles: 18 sleep intervention studies, three experimental sleep manipulation studies, and three studies of anxiety sensitivity. Although the search extended back to 2010, the literature skewed recent, with 14 of the 24 articles published since 2020. Among the 18 sleep intervention studies, nine were randomized controlled trials [22–30] and nine used nonrandomized designs: seven uncontrolled single-group pilot trials [31–37] and two quasi-experimental studies [38, 39]. Fifteen of the 18 intervention studies (83%) reported significant improvements in anxiety [22–25, 27–31, 33, 35–39] and three (16.7%) reported null anxiety findings [26, 32, 34]. Separately, three experimental studies manipulated sleep through restriction or extension [40–42], and three cross-sectional studies measured anxiety sensitivity alongside sleep problems and anxiety [17, 43, 44].

**Table 1:** Study Characteristics

Author Year	Country	Study Design	N	Demographics	Sleep Measure	Anxiety Measure	Key Findings
Sleep interventions							
Akbar et al. (2024) [31]	USA	Non-controlled single-group pilot study (open sleep trial offered to all participants who completed RCT)	46	Age range: 9–14 Age mean: NR % Female: 65%	CSHQ (parent-report); SSR (child-report)	PARS-6 (clinician-rated); SCARED-P	Targeting sleep improvement reduced sleep problems, which mediated reductions in clinician-rated anxiety.
Åslund et al. (2020) [33]	Sweden	Non-controlled single-group pilot study	23	Age range: 13–17 Age mean: 15.5 % Female: 78%	ISI; diagnostic insomnia interview; sleep diary	MINI; SCAS	CBT-I reduced insomnia and produced small - medium (d=0.31) reductions in anxiety.
Åslund et al. (2024) [32]	Sweden	Non-controlled single-group pilot study	27	Age range: 13–17 Age mean: 15.2 % Female: 78%	ISI-a; sleep-wake diary; PDSS	RCADS-C; GAD	CBT-I reduced insomnia. Changes in generalized anxiety scores were not significant and very small.
Baroni et al. (2018) [38]	USA	Controlled quasi-experimental study; sleep course vs. psychopathology course with sleep brochure	145 (sleep 70; comparison 75)	Age range: NR Age mean: 19.8 % Female: 82%	PSQI; ESS; MEQ; SHI; FOSQ; sleep logs	BAI	At 2-month follow-up, sleep-course students reported significantly fewer anxiety symptoms than comparisons.
Bei et al. (2013) [34]	Australia	Non-controlled single-group pilot study; in-school group sleep program	10	Age range: 13–15 Age mean: NR % Female: 100	PSQI; actigraphy	SCAS	The group sleep program improved objective and subjective sleep, but impact on anxiety was minimal.
Blake et al. (2016) [22]	Australia	RCT; sleep intervention vs. active control (study skills)	123 (sleep group 63; control 60)	Age range: 12–16 Age mean: 14.48 % Female: 60%	PSQI; actigraphy; sleep diary	SCAS	Sleep SENSE improved self-reported sleep quality (medium effect), sleep onset latency, daytime sleepiness, objective sleep, and anxiety (small effects).
Cain et al. (2022) [23]	Australia	RCT; sleep restriction vs. bedtime restriction vs. control (time-in-bed regularization)	61	Age range: 6–14 Age mean: 9.1 % Female: 54%	Sleep diary; actigraphy	SCAS; Worry Scale for Children	Both sleep-restriction therapies improved insomnia vs. control. Anxiety and worry improved similarly across all three groups.
Clementi & Alfano (2020) [24]	USA	Pilot RCT; integrated sleep/anxiety treatment vs. standard anxiety CBT	20 (TBT group 10; active control 10)	Age range: 6–12 Age mean: NR % Female: NR	CSHQ; actigraphy	SCARED	Integrated sleep/anxiety treatment produced comparable gains to standard anxiety CBT in both anxiety and sleep.
Cliffe et al. (2020) [35]	UK	Non-controlled single-group pilot study; CBT-I app	39	Age range: 14–17 Age mean: 15.6 % Female: 72%	ISI; SCI; app sleep diaries	RCADS	Digital CBT-I demonstrated pre–post improvements in sleep and anxiety.
de Bruin et al. (2018) [25]	Netherlands	RCT (internet CBT-I, group CBT-I, waitlist)	116	Age range: 12–19 Age mean: 15.6 % Female: 75%	HSDQ; actigraphy	YSR	Internet-delivered CBT-I resulted in decreased anxiety compared to group CBT-I and waitlist. Reduction of insomnia symptoms fully mediated treatment effects on anxiety problems.
Morris et al. (2015) [26]	UK	RCT; 3 groups (“Anxiety Relief,” “Insomnia Relief,” and control)	138 (Anxiety 43; Insomnia 48; control 47)	Age range: NR Age mean: 20 % Female: 62%	PSQI	STAI-S	Those in the Insomnia Relief group showed improved sleep quality but no significant reduction in anxiety symptoms. Those in the Anxiety Relief group showed reduced anxiety symptoms and improved sleep quality.
Okajima et al. (2022) [27]	Japan	RCT; e-mail-delivered CBT-I vs. sleep monitoring	48	Age range: NR Age mean: 19.6 % Female: 67%	ISI; SHPS; DBAS-16; FIRST; PSAS	DASS-21	E-mail-delivered CBT-I improved insomnia and anxiety relative to sleep monitoring, with a significant group effect for anxiety.
Paine & Gradisar (2011) [28]	Australia	RCT; CBT for behavioral insomnia vs. waitlist control	42	Age range: 7–13 Age mean: 9.3 % Female: 43%	Actigraphy; sleep diary; Clinical Sleep History Interview; PDSS	SCAS-C/SCAS-P	SAD, OCD, and total anxiety scores were significantly lower for the CBT group compared to the WL group at post-treatment and 1-month follow-up.
Palermo et al. (2017) [36]	USA	Non-controlled single-group pilot study; brief CBT-I (4 sessions)	40	Age range: 11–18 Age mean: 14.9 % Female: 75%	Actigraphy; sleep diary; ISI; ASWS-R; Adolescent Sleep Hygiene Scale; Pre-Sleep Arousal Scale	CBCL; PROMIS Pediatric Anxiety Short Form	CBT-I reduced anxiety symptoms from pre- to post-treatment and at 3-month follow-up.
Suen et al. (2024) [39]	Hong Kong	Quasi-experimental (matched controls)	274	Age range: 15–24 Age mean: 22.9 % Female: 66%	Sleep quality (one module/outcome)	DASS-21	A multi-component low-intensity online intervention (sleep/relaxation, stress-coping, problem-solving) lowered anxiety symptoms vs. matched controls at 3 months.
Ubara et al. (2022) [29]	Japan	RCT; e-mail-delivered CBTi	45 (CBT-I 24; control 21)	Age range: 18–28 Age mean: 19.7 % Female: 71%	ISI; SHPS; DBAS-16; PSAS	DASS-21	E-mail-delivered CBT-I improved insomnia and anxiety compared to the self-monitoring group.
Werner-Seidler et al. (2019) [37]	Australia	Non-controlled single-group pilot study; CBT-I app	47	Age range: 12–16 Age mean: 13.7 % Female: 66%	ISI; PSQI; sleep diary	GAD-7	App-delivered CBT-I demonstrated pre–post reductions in insomnia symptoms and a small-to-moderate reduction in anxiety.
Yan et al. (2024) [30]	China	RCT; bedtime music therapy (classical vs. jazz vs. control)	75	Age range: 18–39 Age mean: 20.97 % Female: 71%	ISI; PSQI	SAS	Music groups significantly improved insomnia severity and reduced anxiety vs. control.
Experimental sleep restriction or extension studies							
Booth et al. (2021) [40]	Australia	Experimental; manipulated time in bed across sleep restriction (5 h), typical (7.5 h), and sleep extension (10 h) conditions	34	Age range: 15–17 Age mean: 15.91 % Female: 41%	Experimentally manipulated sleep duration	Unipolar visual-analogue mood scales	No statistically significant effect of sleep restriction on fear or anxiety; small-to-moderate effects of restriction to 5–7.5 h were observed on anxiety.
Goldstein et al. (2013) [41]	USA	Experimental; acute total sleep deprivation (24 h) vs. a rested night (~ 8 h)	18	Age range: 18–30 Age mean: 19.6 % Female: 50%	PSQI; sleep diary	STAI; fMRI emotional-anticipation task	Sleep deprivation amplified anticipatory responding during fMRI task. Individuals with the highest trait anxiety showed the greatest neural vulnerability to sleep loss.
Talbot et al. (2010) [42]	USA	Experimental, within-subjects; sleep deprivation (6.5 h first night, 2 h second night) vs. rested (7–8 h for 2 nights)	49	Age range: 10–16 Age mean: 11.5 (early adolescent); 14.29 (mid adolescent) % Female: 50% (early adolescent); 38% (mid adolescent)	Actigraphy; SSS; sleep diary	PANAS-C; anxiety during a catastrophizing task; threat appraisal	Sleep deprivation reduced positive affect and increased anxiety during a catastrophizing task. Early adolescents appraised their main worry as more threatening when sleep-deprived.
Anxiety sensitivity in studies that include both sleep problems and anxiety							
Patriarca et al. (2025) [43]	USA	Cross-sectional	255	Age range: 16–17 Age mean: 11.1 % Female: 58%	CRSP Insomnia Subscale	CASI; ADIS-IV-C/P	Poorer sleep efficiency was significantly correlated with higher anxiety sensitivity. Attentional control accounted for the association between anxiety sensitivity and poor sleep efficiency.
Seehuus et al. (2025) [44]	USA	Cross-sectional	439	Age range: 18–25 Age mean: 20.0 % Female: 48%	SCI	DASS	Insomnia partially mediated the relationship between anxiety sensitivity and state anxiety.
Weiner et al. (2015) [17]	USA	Cross-sectional	101	Age range: 6–17 Age mean: NR % Female: 58%	CSHQ	ADIS-C/P; MASC	Anxiety sensitivity predicted longer sleep onset latency controlling for anxiety. Did not predict night wakings or daytime sleepiness.

*ADIS-C/P* = Anxiety Disorders Interview Schedule for Children/Parents; *BAI* = Beck Anxiety Inventory; *CBT *= Cognitive Behavioral Therapy; *CBT-I* = Cognitive Behavioral Therapy for Insomnia; *CSHQ* = Children’s Sleep Habits Questionnaire; *DASS-21* = Depression Anxiety Stress Scales-21; *ESS *= Epworth Sleepiness Scale; *fMRI *= functional magnetic resonance imaging; *FOSQ *= Functional Outcomes of Sleep Questionnaire; *GAD *= Generalized Anxiety Disorder; *GAD-7 *= Generalized Anxiety Disorder 7-item scale; *ISI *= Insomnia Severity Index; *ISI-a* = Insomnia Severity Index–adolescent version; *MINI *= Mini-International Neuropsychiatric Interview; *NR *= not reported; *ns *= nonsignificant; *PANAS-C *= Positive and Negative Affect Schedule for Children; *PARS *= Pediatric Anxiety Rating Scale; *PROMIS *= Patient-Reported Outcomes Measurement Information System; *PSAS *= Pre-Sleep Arousal Scale; *PSQI *= Pittsburgh Sleep Quality Index; *RCADS *= Revised Children’s Anxiety and Depression Scale; *RCADS-C *= RCADS–Child; *RCT *= Randomized Controlled Trial; *SAD *= Social Anxiety Disorder; *SAS *= Self-rating Anxiety Scale; *SCARED *= Screen for Child Anxiety Related Emotional Disorders; *SCARED-P* = SCARED–Parent; *SCAS *= Spence Children’s Anxiety Scale; *SCAS-P* = Spence Children’s Anxiety Scale–Parent; *SCI* = Sleep Condition Indicator; *SCID *= Structured Clinical Interview for DSM; *SHI *= Sleep Hygiene Index; *SSR *= Sleep Self-Report; *SSS*=Stanford Sleepiness Scale; *STAI *= State-Trait Anxiety Inventory; *YSR *= Youth Self-Report

### Sleep Interventions

The most common sleep intervention was cognitive behavioral therapy for insomnia (CBT-I). In addition to improving sleep problems, clinician-delivered CBT-I reduced anxiety from pre-treatment to post-treatment [28, 33, 36]. Website-based CBT-I [25, 35], email-delivered CBT-I [27, 29], and app-based CBT-I [37] improved both sleep problems and anxiety. Group-based sleep interventions were also effective. A school-based group sleep program for adolescents improved sleep quality [45]. In college students, a semester-long sleep education course significantly reduced anxiety symptoms relative to a comparison group of students enrolled in a child and adolescent psychopathology course who received a sleep hygiene brochure [38]. CBT-I delivered in various forms and dosages resulted in anxiety improvements, most often as a secondary outcome.

Studies that did not include a control group were mostly quasi-experimental or pilot studies, with one exception of a dismantling study. Cain and colleagues [23] found that anxiety improved comparably across all sleep intervention arms. However, every arm received a piece of a sleep intervention, so improvement could not be attributed to a specific component.

Three studies with adolescent samples found null results (16.7%) [26, 32, 34]. For example, in an RCT with separate insomnia-focused and anxiety-focused arms, only the anxiety-focused arm significantly reduced anxiety [26].

### Experimental Sleep Manipulation

Three experimental studies tested the short-term influence of sleep loss on anxiety. Goldstein and colleagues [41] found that sleep deprivation amplified anticipatory neural reactivity in regions central to anxiety in young adults, with the greatest vulnerability among individuals high in trait anxiety. Talbot and colleagues [42] found that sleep deprivation increased anxiety during a worry task for early- and mid-adolescents. Booth and colleagues [40] found that experimental sleep restriction worsened mood but did not significantly change fear or anxiety in adolescents. The experimental evidence supports a causal contribution of sleep loss to anxiety-relevant processes, although effects on anxiety itself were inconsistent.

### Anxiety Sensitivity as an Understudied Mechanism

Anxiety sensitivity is a plausible but underexamined link between sleep and anxiety. Only three studies examined anxiety sensitivity in exclusively pediatric/young adult samples. The three studies were cross-sectional and conducted in the United States. Within the three included studies, higher anxiety sensitivity was associated with sleep problems [17, 43], and insomnia partially accounted for the relationship between anxiety sensitivity and anxiety [44]. No intervention study measured anxiety sensitivity, so whether sleep improvement reduces anxiety by lowering anxiety sensitivity has not yet been tested.

## Discussion

In this systematic review, sleep interventions were effective in improving anxiety in youth. Additionally, a very small literature of experimental studies assigning participants to a specified time in bed supported short sleep duration as a cause of increased anxiety. Sleep problems often precede anxiety in youth, and results of the present review suggest that improving sleep can reduce anxiety across a range of formats and developmental stages. Findings support effectiveness of sleep interventions delivered both in-person and through eHealth, as well as individually and in groups.

Theoretically, sleep loss heightens physiological and affective arousal [46], potentially leading to increased hypervigilance and cognitive rumination [47]. Addressing sleep disturbances may therefore reduce anxiety by lowering arousal. The experimental findings of Goldstein and colleagues [41] and Talbot and colleagues [42] support this notion, as both showed that sleep loss increased anxiety-relevant responding. Anxiety sensitivity, a concept marked by increased attention to and fearful appraisal of one’s internal states, may similarly elevate arousal via increased worries and associated physiological and emotional changes.

### Practical Advantages of Sleep Interventions

Sleep interventions carry practical advantages for anxiety disorder prevention. Stigma may be a barrier for adolescents seeking treatment for anxiety symptoms [6], while sleep tends to be a less stigmatized topic. Interventions framed around improving sleep may be more likely to reach youth who would not accept or engage with anxiety treatment. Further, adolescents in group anxiety/depression treatments may co-ruminate which worsens symptoms [48]. Co-rumination is less likely in a sleep intervention.

### Feasibility of Sleep Interventions in Health Care

Sleep interventions are scalable and can be implemented by many health professionals. Although historically many sleep interventions have been lengthy (e.g., 8–12 in-person sessions), there is increasing support for very brief (1–2 sessions) sleep interventions being effective. Our work has found support for a single session of a cognitive behavioral sleep intervention for children with emotional/behavioral disorders [49] and a community sample of adolescents [50]. Researchers have called for Single Session Interventions to increase the scalability and accessibility of interventions that are often out of reach to many in need [51]. Single Session Interventions have been intentionally and successfully implemented for a range of problems including anxiety [52, 53], depression [52], restrictive eating [52], alcohol use [54], non-suicidal self-injury [55, 56], empathy [57], and pain [58]. Separate meta-analyses indicate Single Session Interventions significantly reduced youth mental health problems (50 RCTs) [59] and sexually transmitted infections (29 interventions) [60] with comparable effect sizes to longer-term and more expensive interventions. Clinicians may consider implementing single session sleep intervention work for sleep and anxiety. Future researchers are encouraged to test the effectiveness of single session sleep interventions.

### Limitations and Future Directions

Several limitations must be noted. The research base includes some small or uncontrolled sleep interventions. Most published findings were significant, in part because null findings are less likely to be published than significant findings, and we did not seek out unpublished work for this review. Many studies measured anxiety *symptoms* rather than diagnosed disorders, and some combined anxiety with depression into a single internalizing disorder outcome. The anxiety sensitivity literature is small, often not specific to youth (i.e., adult-focused), and entirely cross-sectional.

Randomized controlled trials that compare intervention effectiveness at key developmental points would help clarify prevention targets and maximize efficacy. Additional demographic consideration, including the role of sex and socioeconomic status, also warrant further study. Randomized controlled trials that are adequately powered for anxiety outcomes are needed.

## Conclusions

Sleep is a modifiable target that could be implemented for the prevention of pediatric anxiety disorders. Sleep problems reliably precede anxiety from early childhood through young adulthood. The intervention and experimental literature demonstrate that improving sleep generally reduces anxiety across formats (i.e. in-person, group, digital). Anxiety sensitivity may be a promising potential mechanism, although the evidence remains understudied in pediatric samples. Sleep is modifiable and a commonly accepted topic among youth, which makes it a practical foundation for prevention. Improving sleep may be one of the more accessible routes to preventing anxiety disorders.

## Key References

- Akbar et al. (2025). A targeted sleep enhancement program reduced clinician-rated anxiety in youth, with the effect carried by improvement in sleep, supporting sleep as an indirect route to anxiety reduction.
- Suen et al. (2024). A multicomponent low-intensity online program lowered anxiety relative to matched controls, illustrating scalable delivery.
- Patriarca et al. (2025). In youth, sleep problems were associated with higher anxiety sensitivity.

## Data Availability

No datasets were generated or analysed during the current study.
